# Buffalo Medical and Surgical Association

**Published:** 1884-10

**Authors:** Frederick Peterson

**Affiliations:** Secretary


### Buffalo Medical and Surgical Association.

*Regular Meeting, Sept. 2, 1884.*

President, F. W. Bartlett, in the Chair.

The minutes of the last meeting were read and improved.

*Corrigenda*—In Dr. Hartwig's remarks change the name *Siemens* to *Simon;* in Dr. Cronyn's remarks upon tape-worm erase the last sentence.

Dr. William B. Hawkins was elected a member of the society.

No essayist having been announced, a discussion of some of the prevailing diseases took place. The most important points brought before the association were those relative to the intestinal diseases of children and the dietetic treatment to be followed in such cases.

Dr. Charles G. Stockton led the discussion by the following remarks:

"Among prevailing diseases, that which appears most conspicuous is the gastro-enteric disturbance in young children. Since unwholesome nourishment is the most important causative factor in this disorder, it seems to me worth while to call your attention to a diet that, so far as I can learn, is unappreciated by the profession; for either it is not tried, or the trial is unsuccessful, and few use it. I refer to peptonized milk. By peptonized milk I mean two distinct preparations; one prepared with extract of pancreas and soda, the other with pepsin; the first having a marked alkaline reaction, the other slightly acid.

"I began using the alkaline preparation over two years ago, following the suggestions of Fothergill, and Roberts of Manchester, using for a ferment the 'Liquor Pancreaticus;' but of late I have steadily employed the 'Extractum Pancreatis' of Fairchild Bros. & Foster, with gratifying results.

"I think that the formula advertised by the last-named firm contains too much soda for the continued use of the young child. The gastric secretions probably become vitiated, and vomiting or diarrhœa sometimes ensues, after short use. For this reason I have lessened the amount of soda and find that satisfactory peptonization may be had from ten grains of soda with five of 'Extractum Pancreatis,' or even five grains of each, to a pint of milk. The amount of soda and the degree of peptonization must vary in different cases. After a longer or shorter period most little stomachs rebel against even so small an amount of soda; then pepsin should be used as the ferment.

"To properly peptonize milk with pepsin requires more skill and watchfulness than when the extract of pancreas is used. About ten grains of good saccharated pepsin, or the equivalent of other preparations, are necessary to peptonize a pint of milk.

"For several years past I have employed milk thus treated with satisfactory results. Great care is necessary not to allow the lactic acid fermentation to proceed too far, which may be prevented by removing the milk to an ice-chest directly after it is sufficiently peptonized.

"I hesitate to speak as strongly as I feel concerning the importance of this matter to the artificially-fed infants of our cities. For the past year I have had recourse to this method with nearly every infant under my care that was not nursing the breast, and with such success that I commend the practice without reserve. The best plan is to continue with the pancreatin until it disturbs, then for a short interval use the pepsin. Dr. J. Lewis Smith, in a recent number of the *Medical Record*, relates his experience with peptonized milk by 'Extractum Pancreatis,' and gives the plan unstinted praise."

Dr. Hartwig did not understand how the peptonization of milk with pepsin took place. The milk would curdle, and how could any one know exactly when it had become peptonized?

Dr. Cronyn said there was no greater error in dietetics than that of feeding an infant upon the milk of one cow. There was far less danger in using the mixed milk of a large number of cows, for then some of the defects of one would be in a great measure counterbalanced by the production of the healthier animals of the herd. The farmers in the neighborhood of large cities feed their cattle upon the refuse of breweries and distilleries. The milk of a cow at the time of cutting is very unwholesome. By boiling cows' milk, adding limewater and sweetening, we make it very nearly like human milk. The speaker knew a number of families in which the children have been raised upon condensed milk. Upon the whole, condensed milk is better than that of a country cow. It should always be borne in mind that it is very important to distinguish, in children, between gastric and enteric affections. If symptoms of gastric disorder preponderate, we should naturally employ pepsin; if the enteric are uppermost, pancreatin is the proper physiological remedy.

Dr. Coakley stated that he had tried cows' milk properly diluted, sometimes using pepsin, and had not had favorable results. He had also made use of many cereal preparations, but had finally concluded that condensed milk was, after all, the best infant food. The Swiss condensed milk (not the Anglo-Swiss) had, in his hands, proved to be superior to all others. It was successful in a very large percentage of his cases. He had raised his own children upon it.

Dr. Hauenstein told of a patient with œsophageal stricture from carcinoma whom he had fed for four weeks, per rectum, with milk peptonized according to Fairchild. The man was unable to swallow a drop of anything. Five injections of a pint each were given him daily. This sufficed to keep up his strength to the last, when pneumonia (*schluch-pneumonie*) carried him off. He had walked out daily. Injections of milk alone were tried at first, but were not retained. When peptonized, however, they were wholly absorbed by the rectal mucous membrane.

Dr. Strong said he had always used the following method in preparing food for children, and with very good success: Fresh milk from the country was allowed to stand for a short time. The upper third was then removed, added to twice the quantity of boiling water, and then sweetened. He thought this the best plan of all, and, indeed, deemed this preparation even superior to many mothers' milk.

To a question of Dr. Hartwig as to when Dr. Coakley began to feed babes condensed milk, that gentleman responded: "During the first week, or, if necessary, at once."

Dr. Bartlett considered Swiss condensed milk the best artificial infants' food he could obtain. Aside from this, he had also used a mixture of two-thirds milk from a good cow and one-third oatmeal gruel. If the children were constipated, cornmeal could be used instead of oatmeal. He had felt opposed to peptonized milk because it converted the stomach into a place for the cultivation of bacteria.

Dr. Stockton, in closing the discussion upon infantile dietetics, said: "To peptonize milk with pepsin, add ten grains of good saccharated pepsin to a pint of fresh milk; bring this milk to a temperature of 85° F.; watch it carefully, and as soon as the soft curd begins to form, remove to a cool place, whip it up quickly with a fork, strain through a linen cloth, and thereby extract about one-third of the casein. Too high temperature makes a hard curd, and too long peptonization converts too much of the casein.

"As suggested by Dr. Cronyn, by remembering what part of the alimentary tract is affected, and using the pepsin or the pancreatin accordingly, is sometimes found practicable. By extract of pancreas I have tried to peptonize condensed milk, but with poor success. Other trials are to be made.

"No matter if infants appear to thrive when fed with cereal foods, nevertheless they do not. From such foods do we have mal-nutrition and weakened constitutions. Only when they are milk-fed do we find truly vigorous infants, possessed of strong organs and animated spirits, with backs like a sheep's and eyes like an eagle's."

The discussion now turned upon the treatment of the diarrhœas of children, especially of cholera infantum.

Dr. Hartwig said he used a prescription of pulv. ipecac et opii calomel, āā gr. 1-10, with a little pepsin when there was vomiting and purging. He liked Swiss condensed milk very much. It was sterilized by heating to 75° C. in vacuo when manufactured, and he thought it was this which prevented its peptonization, and not the sugar. He believed the peptonization of milk, though a good method, would be impracticable among poor and plain people. They desired some simpler way. It was more important to teach people to feed their children from a cup or spoon and avoid the bottle. In chronic cases of cholera infantum, where there were five or six motions daily, he used salicylic acid.

Dr. Cronyn, in acute cases, prescribed an acid mixture for internal use while the cold swath was most efficient externally. Vegetable astringents proved useful in the chronic form. He thought much depended upon nutrition. Children with less than six or eight teeth should not be given farinaceous food. He objected even to gruel. Barley water might better be used.

Dr. Coakley considered farinaceous food unobjectionable. If there was not sufficient saliva, it would be digested in the small intestine. Therefore, a small amount of farina could not do harm. There was, in his opinion, no remedy equal to the mercurials, such as calomel or mercury with chalk, in incipient cholera infantum.

Dr. Park said real cholera infantum was seldom met with, and our ordinary cases would be better named if they called them entero-colitis.

Dr. Strong had known of no recoveries from cholera infantum without the administration of mercury in some form.

*Voluntary Communications*—Dr. Park exhibited three very interesting specimens of fracture of the neck of the femur. He said he made no distinction between extra and intra-capsular fracture here.

Dr. Hartwig brought a patient before the association with a peculiar jerking of the penis. Some years ago he had had gonorrhœa, and about four years ago this remarkable motion began. There is nothing the matter with his genito-urinary organs beyond this, and this does not disturb him in any way The motion, which several present jocosely called nystagmus penis and penile chorea, is irregular, and not synchronous with the pulse.

Dr. Cronyn suggested that it was a spasmodic neurosis. If it were his case, he would try a small blister over the sacrum, or an electrode in the urethra and one over the pubis. If this caused the jerking to cease, it would at least prove the nervous origin of the affection.

Dr. Peterson stated that he had constituted himself a committee of one to investigate the length of tape-worms, over which there had been some discussion at the previous meeting. He had accepted the invitation of Dr. Hayd to measure the one at his office. This he found eight feet in length. The doctor said the rest of the worm had been thrown away the night before. Tape-worms are easily extended several feet by pulling upon them. The speaker thought that in those cases where neither, the imagination nor the worm was stretched, the presence of several worms in the same intestine might explain the great lengths expelled in individual cases. It is said that from 10 to 40 worms may co-exist in the same patient. He had tabulated, as follows, the lengths in feet given by the various authorities which were at hand:

| | Tænia Solium. | Tænia Mediocanellata. | Bothryocephalus Latus. |
|---|---|---|---|
| W. H. Ransom.... | 7–10 | Larger than T. Solium. | 16–26, Sometimes 60 |
| Dunglison Dict.... | A few feet to 600 | ........ | 180, Enormous. |
| Cobbold.......... | 10–20 | Larger than T. Solium. | 25 |
| Flint............. | 4–35 | ........ | Larger than T. Solium. |
| Aitken........... | 9–35 | Large. | 6–20 |
| Coats............. | 10–12 | 13–24, Stretched. | 16–26 |
| Wagner........... | 6–10 | 10–12 | 16–26 |
| Birch-Hirschfeld... | 6–10 | 10–12 | 16–26 |
| Leuckart.......... | 6–10 | ...... | ...... |
| Davaine.......... | 20–26 | ...... | ...... |
| Hooper.......... | 4–20 | 4–20 | ...... |
| Ziegler........... | 6–10 | 12 | 16–26 |
| Heller (in Ziemsen) | 6–10 | 19 | 16–26 |

It would be seen from this schedule, that the weight of authority and the best modern judgment made the T. solium 6–10 feet, the T. mediocanellata 10–12 feet, and the B. latus (not met with in America) 16–26 feet in length. It was surprising what little conscience was shown by physicians in general with respect to the measurement of tape-worms.

*Miscellaneous Business*—The Secretary announced having received a note from Dr. Tremaine withdrawing his letter of resignation sent some months before.

Dr. Hartwig, chairman of the committee appointed to inquire into the matter of midwives, as suggested in a paper by Dr. Pryor, reported that the committee had met. They had decided to send a circular to physicians in the city, asking for reports of any cases of malpractice by midwives which may have come under their observation.

A motion was carried that the committee continue its work. The meeting then adjourned.

FREDERICK PETERSON, *Secretary.*

